# Longitudinal associations of active commuting with wellbeing and sickness absence

**DOI:** 10.1016/j.ypmed.2015.12.010

**Published:** 2016-03

**Authors:** Oliver Tristan Mytton, Jenna Panter, David Ogilvie

**Affiliations:** MRC Epidemiology Unit & UKCRC Centre for Diet and Activity Research (CEDAR), University of Cambridge School of Clinical Medicine, Box, 285, Cambridge Biomedical Campus, Cambridge CB2 0QQ, UK

**Keywords:** Adult, Health Status, Psychological, Motor Activity, Walking, Transportation, Absenteeism, Epidemiology, Sick Leave, Bicycling

## Abstract

**Objective:**

Our aim was to explore longitudinal associations of active commuting (cycling to work and walking to work) with physical wellbeing (PCS-8), mental wellbeing (MCS-8) and sickness absence.

**Method:**

We used data from the Commuting and Health in Cambridge study (2009 to 2012; n = 801) to test associations between: a) maintenance of cycling (or walking) to work over a one year period and indices of wellbeing at the end of that one year period; and b) associations between change in cycling (or walking) to work and change in indices of wellbeing. Linear regression was used for testing associations with PCS-8 and MCS-8, and negative binomial regression for sickness absence.

**Results:**

After adjusting for sociodemographic variables, physical activity and physical limitation, those who maintained cycle commuting reported lower sickness absence (0.46, 95% CI: 0.14–0.80; equivalent to one less day per year) and higher MCS-8 scores (1.50, 0.10–2.10) than those who did not cycle to work. The association for sickness absence persisted after adjustment for baseline sickness absence. No significant associations were observed for PCS-8. Associations between change in cycle commuting and change in indices of wellbeing were not significant. No significant associations were observed for walking.

**Conclusions:**

This work provides some evidence of the value of cycle commuting in improving or maintaining the health and wellbeing of adults of working age. This may be important in engaging employers in the promotion of active travel and communicating the benefits of active travel to employees.

## Highlights

• Maintenance of cycling to work was associated with reduced sickness absence.
• This association persisted after adjustment for baseline sickness absence.
• Maintenance of cycling to work was also associated with better mental wellbeing.
• No significant associations were observed for walking to work.

## Introduction

Research on the associations between active travel and health has focused on major diseases and mortality(Jarrett et al., 2012, Laverty et al., 2013, Saunders et al., 2013). In contrast relatively little work has explored the associations between active travel and other measures such as sickness absence(Hendriksen et al., 2010) and wellbeing,(Gómez et al., 2013, Humphreys et al., 2013, Martin et al., 2014, Mutrie, 2002) despite the existence of positive associations between overall physical activity and these outcomes(Amlani and Munir, 2014, Bize et al., 2007, Ferrie et al., 2005, Hendriksen et al., 2010, Laaksonen et al., 2009, Lahti et al., 2012, Proper et al., 2006).

These associations are of interest for several reasons. Wellbeing is important to individuals, and is increasingly recognised as important for governments(Boorman, 2009, Office for National Statistics, 2011). Sickness absence is an important measure for employers,(Office for National Statistics, 2014) and is also a good predictor of future disability or death(Kivimäki et al., 2004, Kivimäki et al., 2003, Marmot et al., 1995). If either measure were shown to be associated with active travel, this might strengthen the case for employers investing in its promotion(Black, 2008, National Institute for Health and Clinical Excellence, 2012, National Institute for Health and Clinical Excellence, 2008). These measures may also be more sensitive to change than disease end points in a relatively healthy population of working age, and therefore may be appropriate outcomes to use in some studies of the effect of active travel on health.

Research in this area has also frequently been limited to cross-sectional studies (Gómez et al., 2013, Hendriksen et al., 2010, Humphreys et al., 2013) which provide a weak basis for inferring causation. Some studies present conflicting findings, particularly concerning the association between active travel and mental well-being (Gómez et al., 2013, Humphreys et al., 2013, Martin et al., 2014, Mutrie, 2002). In this study, we build on previous cross-sectional analysis using data from the Commuting and Health in Cambridge study, which explored the associations between active commuting and wellbeing(Humphreys et al., 2013). With the addition of follow-up data from the same cohort, our aim in this paper is to explore the longitudinal associations of active commuting with physical wellbeing, mental wellbeing and sickness absence.

## Methods

### Study setting and data collection

The analysis used data from the Commuting and Health in Cambridge study, a longitudinal study of commuters working in Cambridge, UK (n = 1431). A full description of this study has been published elsewhere(Ogilvie et al., 2010). Participants completed up to four annual questionnaires (2009–2012) that included information on travel behaviour, physical activity, sociodemographic characteristics and measures of health and wellbeing. Ethical approval was granted by the Hertfordshire Research Ethics Committee and the Cambridge Psychology Research Ethics Committee. All participants gave written informed consent.

### Inclusion and exclusion criteria

New participants were recruited during each of the first three years of the study. As only a small number of participants completed three or four waves of the study, we restricted our analysis to those who completed two consecutive waves of the study (n = 866). We further excluded those with missing exposure (n = 25), outcome (n = 5) or covariate data (n = 35), such that we undertook a complete case analysis (n = 801). We defined the baseline questionnaire for each participant as their first questionnaire with complete information on exposure. The follow-up questionnaire for each participant was the questionnaire completed one year after their baseline questionnaire.

### Exposure measures

The primary exposures of interest were maintenance of cycling to work and maintenance of walking to work. While these exposures were ascertained at baseline for each participant, we chose to restrict our analysis to those who were confirmed at follow-up to have comparatively stable commuting behaviour. This ensured that estimates of association would not be influenced by the potential misclassification of those who changed their behaviour during the period of observation (e.g. if a participant switched from cycling to work to not cycling to work two weeks after baseline data collection). The secondary exposures of interest were change in weekly time spent cycling to work and change in weekly time spent walking to work.

Weekly time spent cycling to work at each time point was estimated by summing the total number of trips to and from work involving any cycling that were reported in a seven day travel record, and multiplying this by the typical duration of cycling per trip (assessed in a separate question) (Panter et al., 2011). Maintenance of cycling to work was defined as weekly cycling time > 0 minutes at both baseline and follow-up. The reference group consisted of those who did not cycle to work at both baseline and follow-up. Consequently participants who stopped cycling to work (e.g. weekly cycling time > 0 minutes at baseline and weekly cycling time = 0 minutes at follow-up) or took up cycling to work were not categorised, and were therefore excluded from analyses that used this exposure measure.

Change in weekly time cycling to work between baseline and follow-up was categorised as either any increase, no change, or any decrease, based on the difference in the estimates of time cycling to work at baseline and follow-up. As small increases or decreases might reflect reporting errors rather than true changes, we also conducted a sensitivity analysis in which only large increases or decreases in cycle commuting time (≥ 50 min/week) were categorised as ‘change’, and smaller changes were re-categorised as ‘no-change’(Panter et al., 2015).

The same process was followed for walking to work.

### Outcome measures

We used three measures (physical wellbeing, mental wellbeing and sickness absence), hereafter collectively referred to as “indices of wellbeing”. Physical Component Summary (PCS-8) and Mental Component Summary (MCS-8) scores were derived from responses to the Medical Outcomes Study Short Form questionnaire (SF-8) (see appendix) (Ware et al., 2001). The SF-8 questionnaire comprises eight ordinal response questions concerning participants’ wellbeing in the past four weeks, with different weights being applied to each question to derive the scores as described by Ware et al (Ware et al., 2001). In our analysis the two scores were treated as continuous variables and analysed as separate outcomes, as one might expect each measure to have different associations with active travel (Humphreys et al., 2013, Richards et al., 2015). Sickness absence was self-reported as the total number of days absent from work in the past year, using a validated question (Ferrie et al., 2005).

### Covariates

Date of birth, date of questionnaire completion, education, sex, height, weight, difficulty walking, limitation of physical activity, home postcode, home to work distance, and physical activity (Recent Physical Activity Questionnaire)(Besson et al., 2010) were assessed by questionnaire. Dates of birth and questionnaire completion were used to calculate age. Weight status (low or healthy weight, overweight, obese) was assigned based on participant’s body mass calculated by dividing weight by height squared (World Health Organisation, 2000). Physical activity level (inactive, moderately inactive, moderately active, active) was assigned based on occupation and time spent in recreational activity following the Cambridge Physical Activity Index (Wareham et al., 2003). While the original index incorporated walking and cycling to work, we excluded time spent in these activities when assigning participants. A physical limitation variable (yes/no) was created, with participants being assigned to ‘yes’ if they either (a) reported difficultly walking for a quarter of a mile on the level or (b) reported that physical health problems limited their ability to do usual physical activities.

### Analysis

We used two complementary approaches to testing longitudinal associations.

In the first set of analyses, we modelled the associations between maintenance of cycling (or walking) to work and indices of wellbeing at follow-up. These ‘maintenance analyses’ were intended to contribute to establishing evidence of a temporal relationship, because the exposure was ascertained before the outcome (Hill, 1965). We used linear regression to test the associations of maintenance of cycling (or walking) to work with PCS-8 and MCS-8. However, sickness absence was positively skewed with a large number of zero counts. Following Zhou et al,(Zhou et al., 2014) we fitted different models (e.g. linear, binomial, negative binomial, zero-inflated) and found our data were fitted best by a negative binomial distribution. Consequently we used negative binomial regression to test the associations with sickness absence. Regression models were adjusted for all covariates (age, sex, education, physical activity, weight status, physical limitation, home-work distance and study year) (model A).

We further conditioned each analysis on the baseline value of the outcome variable in question (i.e. analysis of covariance) (model B). In this context, analysis of covariance addresses whether there is a difference in the change in outcome between cyclists and non-cyclists *who have the same initial value of the outcome*? It is considered the most appropriate approach to test for differences in change between two groups, when there are baseline differences in the outcome of interest between groups (Fitzmaurice, 2001, Twisk and Proper, 2005).

In the second set of analyses, we used linear regression to test the associations between change in cycling (or walking) to work and changes in indices of wellbeing. By focusing on individuals who changed their behaviour, these ‘change analyses’ were intended to provide a more direct estimate of the effect that might be induced by increasing or reducing a given behaviour. Change in sickness absence had a positive kurtosis, and we truncated outliers (to +/− 30 days) so that residuals were normally distributed. We used the same approaches to adjustment for covariates described above (model A and model B).

In summary we used two analytic approaches (‘maintenance’ and ‘change’), each with two stages of adjustment for covariates (model A and model B), applied to two exposures (cycling and walking to work) and three outcomes (PCS-8, MCS-8 and sickness absence). We also undertook sensitivity analyses adjusting the ‘maintenance’ analyses for the reciprocal commuting behaviour (e.g. models using cycling to work as the exposure were additionally adjusted for walking to work). All analyses were conducted in Stata v13.

## Results

The participants included in analysis were predominantly women (69.7%), educated to degree level or higher (70.2%), of low or healthy bodyweight (65.4%), and slightly more than half reported cycling to work (54.3%) (Table 1). The average scores for physical wellbeing (median PCS-8 55.5, IQR 51.5 to 58.0) and for mental wellbeing (median MCS-8 52.5, IQR 48.5 to 57.5) were higher than the specified population average (50). Sickness absence (mean = 3.6 days, median 1 day, IQR 0 to 4 days) was lower than the UK mean (4.4 days) (Office for National Statistics, 2014). Baseline differences between those who cycled to work and those who did not (and the equivalent for walking) are shown in Table 1, and differences between participants included in and excluded from the analysis are shown in Table A1.

### Cycling maintenance and wellbeing

In univariable analysis, those who maintained cycling to work were found to report higher PCS-8 and MCS-8 scores at follow-up than those who did not cycle to work (Table 2). For PCS-8 the association was no longer significant after adjustment for covariates (model A), although the effect size estimate was of similar magnitude. The association between maintenance of cycling to work and MCS-8 remained significant after adjustment for covariates (model A), but not after additional adjustment for baseline MCS-8 (model B). Sensitivity analysis, adjusting for walking to work, resulted in a similar pattern and magnitude of findings, although the only significant finding was for PCS-8 (model A: 1.45, 95% CI 0.06 to 2.84; n = 573).

### Cycling maintenance and sickness absence

Maintenance of cycling to work was associated with reduced sickness absence in univariable analysis (Table 3). The association remained after adjustment for covariates (model A) and baseline sickness absence (model B). The effect size (0.5) was equivalent to just over one day of sickness absence per year, and was similar in univariable and adjusted analyses.

### Changes in weekly cycling time and changes in indices of wellbeing

There were no significant associations between either any change, or a large change, in weekly cycling time and changes in PCS-8, MCS-8 or sickness absence (Tables 4; A5). However, the adjusted effect sizes for PCS-8 (model B) and MCS-8 (model B) in these change analyses were comparable to those observed in the maintenance analyses (PCS-8: 1.01 vs 0.87; MCS-8: 0.69 vs 0.82).

### Walking

There were no significant associations between maintenance of walking and PCS-8, MCS-8 or sickness absence (Tables A2 and A3), or between change in walking and change in any of these outcomes, in either the main or the sensitivity analyses (Tables A4, A6).

## Discussion

### Principal findings

We found that maintenance of cycling to work was associated with reduced sickness absence during the year of follow-up, after adjustment for covariates and baseline sickness absence. Taken together our results are consistent with cycling to work being important for both mental and physical wellbeing. We did not find any evidence of associations between walking to work and any of the indices of wellbeing.

### Strengths and limitations

The primary strength of this study lies in the use of complementary longitudinal analyses to test the little-studied associations of active commuting with sickness absence and wellbeing. The outcomes (indices of wellbeing) were measured after the exposure (active commuting), which is important for building a case for causal associations. Nonetheless we should be cautious in our interpretation, particularly given the plausibility of a bi-directional relationship between active travel and these indices.

Although, this study may appear comparatively small and thereby lack power compared to some cohort studies, it is larger than some other studies focusing on active travel and health, and consequently makes an important contribution (MacDonald et al., 2010, Mutrie, 2002). While it relies on self-reported measures of exposure and outcome, this is entirely appropriate for wellbeing (which depends on self-report) and appears unlikely to have resulted in important biases for the other measures. We have previously shown good agreement between self-reported and objective estimates of time spent in active commuting using this dataset (Panter et al., 2014) and the measure of self-reported sickness absence has been shown to have good agreement with employer records of sickness absence in another UK cohort (Ferrie et al., 2005). Nonetheless, because we used data from a study designed to investigate the relationships between commuting and health, it is possible that responses might have been influenced by social desirability bias. While such a bias could account for differences in wellbeing between those who did and did not use active modes of travel, it is unclear why this might have occurred only for cycling and not for walking. The study population was relatively affluent, educated and predominantly white-collar,(Goodman et al., 2012, Humphreys et al., 2013) so the findings may not be readily generalizable to other settings.

### Comparison with other studies

Concerning wellbeing, our findings broadly agree with previous work (Humphreys et al., 2013, Martin et al., 2014, Mutrie, 2002). In a randomised controlled trial of an intervention that increased walking to work, Mutrie et al. found improvements in three of eight sub-scales of wellbeing assessed using the SF-36. Because they used the SF-36 and did not compute MCS and PCS scores, direct comparisons with our study are not possible, but we note that mental health was one of the three domains in which they observed significant improvements, paralleling the increase in MCS-8 that we observed for cycling. In a large longitudinal survey of British adults, Martin et al. found that active commuting, compared to car use, was associated with better psychological wellbeing assessed using the General Health Questionnaire (GHQ-12) (Martin et al., 2014). While our findings may appear to differ from those of our previous cross-sectional analysis, which found a positive association between active commuting and physical wellbeing and a null association for mental wellbeing, the direction and magnitude of the effects observed in the two analyses are not necessarily inconsistent. In the previous analysis, the exposure was categorised differently, with active commuting being defined as ‘any walking or cycling to work’ (Humphreys et al., 2013). The analyses reported here used two separate exposures, cycling to work and walking to work, for which we found associations with MCS-8 (albeit not always significant) in opposite directions. This suggests that the null association between active commuting and MCS-8 reported previously could reflect a combination of positive associations for cycling and negative associations for walking. The positive associations between active commuting and wellbeing are also consistent with a much broader evidence base showing positive associations between physical activity (typically leisure-time physical activity) and wellbeing among healthy adults, as well as our own analyses (see ‘physical activity index’ in Tables 2 and A2) (Bize et al., 2007).

Our findings contrast with those of one study, of women living in low and middle income neighbourhoods in the urban area of Cali, Colombia (Gómez et al., 2013). That study observed negative associations of walking for transport with both PCS-8 and MCS-8, whereas we observed a null association for PCS-8 and a non-significant (although negative) association for MCS-8. Interpreting our results in the context of the Colombian results might appear to give more plausibility to a negative association between walk commuting and mental wellbeing. Nonetheless there are important compositional differences between the two studies. For example, our study in Cambridge predominantly included comparatively affluent participants. Some commuters in Cambridge, in part because of their financial resources, can exert choice over their mode of travel to work, and if travel mode is chosen rather than constrained, then active travel may be experienced as being more pleasant (Goodman et al., 2012). There are also different contextual factors. For example, walking for transport may be more associated with activities perceived as burdensome, or concerns about personal safety, in Cali than in Cambridge.

Concerning sickness absence, our findings are very similar to those of Hendriksen et al., who found that cycling to work was associated with just over one fewer day of employer-recorded sickness absence per year in a sample of Dutch workers (Hendriksen et al., 2010). Their analysis was in a population with a higher level of sickness absence (mean = 8 days) than ours (3.6 days). It was also cross-sectional and consequently was not adjusted for baseline sickness absence, although it was adjusted for self-reported health and measures of chronic disease. Our findings are also consistent with a broader literature suggesting positive associations between physical activity (again, predominantly recreational) or physical fitness and sickness absence (Amlani and Munir, 2014, Lahti et al., 2012).

### Interpretation and Implications

Taken together our findings provide some evidence that cycling to work may contribute to better wellbeing and reduced sickness absence. The effect size we observed for PCS-8 is similar to the differences we observed in our cohort between the young (16–29 years) and old (> 50 years), or between those with obesity and those of low or healthy bodyweight. The effect size for MCS-8 is similar to that which we observed between men and women in our cohort.

For both the ‘maintenance’ and the ‘change’ analyses we estimated two sets of models, one adjusting for covariates, and a second additionally conditioning on the baseline value of the outcome in question. The pattern of results for MCS-8 (significant after adjustment for covariates, not significant after additionally conditioning on baseline MCS-8) suggests that the differences observed between those who cycled to work and those who did not are, at least partly, due to differences in MCS-8 between the two groups at baseline. In contrast, the equivalent models for sickness absence produced effect estimates that were both statistically significant and similar in magnitude to each other. This suggests that even after allowing for differences in sickness absence between those who cycled to work at baseline and those who did not, the cyclists were still likely to report less sickness absence at follow-up (Fitzmaurice, 2001).

In light of the positive findings for cycling to work, the null findings for walking to work may appear unexpected. It seems unlikely that they can be explained simply by many non-walkers cycling to work, as additional adjustment for cycling to work did not reveal any significant associations. Among those who reported walking to work in our study, the average weekly duration of time walking was relatively low compared to other studies and to estimates of walking undertaken by the average office worker at work (Clemes et al., 2014). This partly reflects our survey, which was designed to capture *any* walking on the commute and therefore includes short walking journeys as part of a longer journey (e.g. by public transport) as well as trips made entirely by foot. We also note that walking is undertaken at a lower intensity than cycling(Ainsworth et al., 2011, Costa et al., 2015) and that intensity of physical activity can be an important determinant of an activity’s effect on health. This may be particularly true for sickness absence, for which only vigorous but not moderate physical activity has been associated with reduced absence (Lahti et al., 2012). It is possible then that the average ‘dose’ of walking to work in the exposed group in our study was too low for effects on wellbeing and sickness absence to be observed. Other differences between cycling and walking in Cambridge for example in relation to motivations, perceptions as well as psychological and social considerations(Guell et al., 2013, Guell et al., 2012) may also have contributed to the different patterns of association with indices of wellbeing.

Finally, our findings also provide indicative estimates of effect sizes that might be observed in future studies of the health benefits of interventions to promote active travel, which may form the basis for power calculations.

### Unanswered questions and future research

Considerable uncertainty remains concerning the dose, frequency and intensity of active travel necessary to realise sickness absence and wellbeing benefits. Future research should seek to reduce this uncertainty and test more thoroughly whether changes in travel behaviour are associated with changes in wellbeing or sickness absence. It should also seek to replicate our findings in different populations, particularly in more deprived communities in which commuting choices may be more constrained and active travel perceived differently. Exploring the differential benefits of active travel among those who are obese or sedentary would also be of value as they may have more to gain (Ekelund et al., 2015).

## Conclusions

Our work provides some evidence of the potential contribution of cycle commuting to reducing sickness absence and improving or maintaining wellbeing in adults of working age. Our findings may be important for employers seeking to reduce sickness absence, to governments seeking to adopt policies to promote wellbeing, and to individuals choosing how to travel to work.

## Conflict of interest

The authors declare there is no conflict of interest.

## Transparency document

Transparency document.

## Figures and Tables

**Table 1:** Characteristics of participants included in the analyses (n = 801).

	Cycling to work at baseline		Walking to work at baseline	
	None (n = 366)	Some (n = 435)	None (n = 597)	Some (n = 204)
	N (%)	N (%)	N (%)	N (%)
*Gender*				
Female	283 (50.7)	275 (49.3)	404 (72.4)	154 (27.6)
Male	83 (34.2)	160 (65.8)	193 (79.4)	50 (20.6)

*Age*				
Median	44.4 (34.7–52.9)	43.0 (33.1–51.6)	43.2 (34.0–52.0)	43.3 (33.7–52.2)
16–29 years	42 (39.6)	64 (60.4)	73 (68.9)	33 (31.1)
30–39 years	103 (46.8)	117 (53.2)	169(76.8)	51 (23.2)
40–49 years	93 (44.3)	117 (55.7)	160 (76.2)	50 (23.8)
50–59 years	92 (46.2)	107 (53.8)	148 (74.4)	51 (25.6)
≥ 60 years	36 (54.6)	30 (45.5)	47 (71.1)	19 (28.8)

*Highest educational qualification*				
Less than degree	140 (58.6)	99 (41.4)	177 (74.1)	62 (25.9)
degree or higher	226 (40.2)	336 (59.8)	420 (74.5)	142 (25.3)

*Deprivation quintile*				
1 (= least deprived)	181 (49.7)	183 (50.3)	269 (73.9)	95 (26.1)
2	110 (46.8)	125 (53.2)	180 (76.6)	55 (23.4)
3	50 (48.1)	54 (51.9)	71 (68.3)	33 (31.8)
4	22 (23.9)	70 (76.1)	73 (79.3)	19 (20.7)
5 (= most deprived)	3 (50.0)	3 (50.0)	4 (66.7)	2 (33.3)

*Weight status*				
low or healthy weight	213 (40.6)	311 (59.4)	395 (75.4)	129 (24.6)
Overweight	102 (49.8)	103 (50.2)	148 (72.2)	57 (27.8)
Obese	51 (70.8)	21 (29.2)	54 (75.0)	18 (25.0)

PCS-8 score				
Median (IQR)	55.2 (51.1–58.0)	55.7 (52.2–58.0)	55.5 (51.7–58.0)	55.4 (51.6–58.0)

*MCS-8 score*				
Median (IQR)	52.3 (47.1–57.5)	52.6 (49.4–57.5)	52.5 (49.2–57.5)	52.3 (47.3–57.5)

*Sickness Absence (days per year)*				
Median (IQR)	2 (0–4)	1 (0–3)	1 (0–3)	2 (0–5)

*Physical limitation*				
Yes	350 (45.4)	421 (54.6)	575 (74.6)	196 (25.4)
No	16 (53.3)	14 (46.7)	22 (73.3)	8 (26.7)

*Home to work distance*				
Median (IQR)	19.3 (8.0–27.4)	4.8 (3.2–8.1)	8.0 (4.8–19.3)	8.0 (3.2–24.1)
0–9.99 km	117 (24.8)	354 (75.2)	361 (76.7)	110 (23.4)
10–19.99 km	71 (60.7)	46 (39.3)	89 (76.1)	28 (23.9)
≥ 20 km	178 (83.6)	35 (16.4)	147 (69.0)	66 (31.0)

*Physical activity index*				
Inactive	9 (37.5)	15 (62.5)	19 (79.2)	5 (20.8)
Moderately inactive	111 (50.5)	109 (49.5)	162 (73.6)	58 (26.4)
Moderately active	113 (46.1)	132 (53.9)	180 (73.5)	65 (26.5)
Active	133 (42.6)	179 (57.4)	236 (75.6)	76 (24.4)

*Weekly time cycling to work (minutes)*				
Median (IQR)	0 (0–0)	150 (90–200)	100 (0–180)	0 (0–45)

*Weekly time walking to work (minutes)*				
Median (IQR)	0 (0–90)	0 (0–0)	0 (0–0)	100 (60–180)

*Changed behaviour*				
Started walking/cycling to work	42 (9.7)	0 (0)	75 (36.7)	0 (0)
Stopped walking/cycling to work	0 (0)	68 (18.6)	0 (0)	67 (11.2)

*Time frame*				
2009–10 (reference)	305 (47.0)	344 (53.0)	477 (73.5)	172 (26.5)
2010–11	16 (39.0)	25 (61.0)	25 (61.0)	16 (39.0)
2011–2	45 (40.5)	66 (59.5)	95 (85.6)	16 (14.4)

IQR = Interquartile range; PCS-8 = Physical Component Summary score derived from the Short Form 8 Questionnaire, theoretical score range is 9.1 to 69.0, with a mean of 50 in the US adult population; MCS-8 = Mental Component Summary score derived from the Short Form 8 Questionnaire, theoretical score range is 5.4 to 71.7, with a mean of 50 in the US adult population; deprivation quintile is based on national quintiles of deprivation ranked using the Index of Multiple Deprivation 2010 score for the Lower Super Output Area (assigned based on postcode or residence); unless otherwise stated characteristics are measured at baseline; changed behaviour describes the number of individuals who started or stopped active travel between baseline and follow-up (e.g. cycle to work at baseline and not cycling to work at follow-up); Study undertaken in Cambridge, UK (2009-12).

**Table 2:** Associations between maintenance of cycling to work and wellbeing (n = 691).

		Physical Wellbeing (PCS-8)			Mental Wellbeing (MCS-8)		
		Unadjusted	Model A	Model B	Unadjusted	Model A	Model B
		Coefficient (95% CI)	Coefficient (95% CI)	Coefficient (95% CI)	Coefficient (95% CI)	Coefficient (95% CI)	Coefficient (95% CI)
Cycling	None (reference)						
	Some	**1.05 (0.13, 1.96)**	1.08 (-0.06, 2.23)	0.87 (-0.17, 1.93)	**1.33 (0.19, 2.48)**	**1.50 (0.10, 2.90)**	0.82 (-0.42, 2.08)
Gender	Male (reference)						
	Female	0.24 (-0.76, 1.25)	0.31 (-0.72, 1.24)	0.14 (-0.81, 1.09)	**-1.81 (-3.06, -0.56)**	-1.14 (-2.41, 0.12)	-0.95 (-2.09, 0.17)
Age	16-29 years (reference)						
	30-39 years	-0.37 (-1.97, 1.21)	-0.24 (-1.85, 1.36)	-0.12 (-1.59, 1.35)	**2.43 (0.47, 4.39)**	**2.64 (0.68, 4.61)**	**1.83 (0.07, 3.58)**
	40-49 years	-0.33 (-1.92, 1.26)	-0.26 (-1.86, 1.35)	-0.07 (-1.54, 1.41)	**3.92 (1.96, 5.88)**	**3.88 (1.91, 5.85)**	**3.06 (1.30, 4.82)**
	50-59 years	-1.25 (-2.84, 0.35)	-1.09 (-2.72, 0.54)	-0.73 (-2.23, 0.75)	**3.74 (1.77, 5.71)**	**3.67 (1.68, 5.66)**	**2.69 (0.91, 4.48)**
	≥ 60 years	-1.30 (-3.33, 0.74)	-0.91 (-2.98, 1.17)	-0.68 (-2.58, 1.21)	**5.49 (2.98, 7.99)**	**5.87 (3.33, 8.40)**	**3.72 (1.44, 6.05)**
Degree	No (reference)						
	Yes	0.14 (-0.85, 1.15)	-0.38 (-1.46, 0.70)	-0.73 (-1.73, 0.25)	0.03 (-1.22, 1.29)	-0.27 (-1.59, 1.05)	-0.23 (-1.40, 0.94)
Home to work distance	0-9.99 km (reference)						
	10-19.99 km	-0.10 (-1.43, 1.22)	0.54 (-0.88, 1.98)	0.47 (-0.84, 1.77)	0.07 (-1.59, 1.72)	0.04 (-1.71, 1.78)	0.35 (-1.20, 1.54)
	≥ 20 km	-0.60 (-1.66, 0.46)	0.13 (-1.18, 1.44)	-0.01 (-1.21, 1.19)	-0.46 (-1.79, 0.86)	0.23 (-1.37, 1.82)	0.54 (-0.88, 1.97)
Physical limitation	No (reference)						
	Yes	-**4.10 (-6.55, -1.66)**	**-3.84 (-6.33, -1.35)**	**4.03 (1.39, 6.68)**	**-4.30 (-7.36, -1.23)**	**-3.81 (-6.86, -0.77)**	**-3.16 (-5.88, -0.45)**
Physical Activity	Inactive (reference)						
	Moderately inactive	0.73 (-2.10, 3.57)	0.41 (-2.47, 3.29)	-1.14 (-3.79, 1.49)	**4.33 (0.81, 7.86)**	**4.96 (1.45, 8.48)**	2.68 (-0.47, 5.83)
	Moderately active	1.57 (-1.26, 4.40)	1.14 (-1.76, 4.03)	-0.81 (-3.49, 1.85)	**4.37 (0.86, 7.88)**	**5.09 (1.54, 8.06)**	2.58 (-0.58, 5.76)
	Active	1.53 (-1.26, 4.34)	1.02 (-1.84, 3.89)	-0.77 (-3.41, 1.87)	**5.58 (2.10, 9.06)**	**6.00 (2.49, 9.50)**	2.88 (-0.28, 6.03)
Weight status	Low or healthy (reference)						
	Overweight	-0.68 (-1.75, 0.39)	0.56 (-1.39, 0.82)	-0.30 (-1.32, 0.71)	-0.25 (-1.60, 1.09)	-0.37 (-1.72, 0.98)	-0.39 (-1.60, 0.81)
	Obese	**-1.72 (-3.35, 0.09)**	-1.11 (-2.82, 0.59)	-0.51 (-2.08, 1.05)	0.29 (-1.76, 2.33)	0.80 (-1.29, 2.89)	0.47 (-1.39, 2.34)
Study Year	2009-10 (reference)						
	2010-11	0.05 (-2.09, 2.19)	-0.29 (-2.44, 1.85)	-0.11 (-2.08, 1.84)	1.14 (-1.52, 3.81)	1.14 (1.47, 3.76)	0.13 (-2.20, 2..47)
	2011-2	-0.74 (-2.10, 0.60)	-1.08 (-2.47, 0.32)	-0.86 (-2.14, 0.41)	-0.06 (-1.74, 1.62)	-0.06 (-1.76, 1.65)	-0.42 (-1.94, 1.10)
Baseline wellbeing		**0.42 (0.36, 0.50)**		**0.48 (0.40, 0.56)**			**0.46 (0.39, 0.53)**

Linear regression coefficients shown; CI = confidence interval; PCS-8 = Physical Component Summary score derived from the Short Form 8 Questionnaire; MCS-8 = Mental Component Summary score derived from the Short Form 8 Questionnaire; physical activity is categorised using the Cambridge Physical Activity Index; weight status is categorised using body mass index; study year refers to the time period when data were collected; Model A is adjusted for gender, age, degree, home to work distance, physical limitation, physical activity, weight status and study year; Model B is adjusted for gender, age, degree, home to work distance, physical limitation, physical activity, weight status, study year and baseline wellbeing (baseline PCS-8 for PCS-8 model or baseline MCS-8 for MCS-8 model); bold indicates significant results (p < 0.05); Study undertaken in Cambridge, UK (2009-12).

**Table 3:** Association between cycling to work and sickness absence (n = 691).

		Unadjusted	Model A	Unadjusted
		Co-efficient (95% CI)	Co-efficient (95% CI)	Co-efficient (95% CI)
Cycling	None (reference)			
	Some	**-0.51 (-0.76, -0.26)**	**-0.47 (-0.80, -0.14)**	**-0.46 (-0.77, -0.14)**
Gender	Male (reference)			
	Female	0.18 (-0.10, 0.46)	0.12 (-0.14, 0.40)	0.17 (-0.10, 0.43)
Age	16-29 years (reference)			
	30-39 years	**-0.42 (-0.85, 0.02)**	**-0.50 (-0.93, -0.06)**	**-0.43 (-0.84, -0.01)**
	40-49 years	**-0.79 (-1.22, -0.35)**	**-0.88 (-1.31, -0.44)**	**-0.80 (-1.21, -0.37)**
	50-59 years	-0.27 (-0.70, 0.14)	**-0.44 (-0.89, 0.00)**	-0.42 (-0.85, 0.01)
	≥ 60 years	**-0.56 (-1.11, -0.02)**	**-0.79 (-1.35, -0.22)**	**-0.56 (-1.10, -0.02)**
Degree	No (reference)			
	Yes	**-0.34 (-0.62, -0.07)**	-.10 (-0.39, 0.19)	-0.01 (-0.29, 0.27)
Home to work distance	0-9.99 km (reference)			
	10-19.99 km	0.14 (-0.21, 0.50)	-0.10 (-0.50, 0.29)	-0.09 (-0.47, 0.28)
	≥ 20 km	**0.38 (0.09, 0.66)**	-0.07 (-0.44, 0.29)	-0.12 (-0.47, 0.22)
Physical limitation	No (reference)			
	Yes	**1.22 (0.59, 1.86)**	**0.97 (0.34, 1.61)**	0.51 (-0.11, 1.13)
Physical Activity	Inactive (reference)			
	Moderately inactive	-0.19 (-0.96, 0.57)	-0.02 (-0.78, 0.74)	0.70 (-0.08, 1.48)
	Moderately active	-0.11 (-0.88, 0.65)	-0.04 (-0.81, 0.73)	0.72 (-0.07, 1.51)
	Active	-0.33 (-1.09, 0.43)	-0.08 (-0.86, 0.68)	0.54 (-0.25, 1.33)
Weight status	Low or healthy (reference)			
	Overweight	0.13 (-0.16, 0.42)	0.17 (-0.12, 0.47)	0.18 (-0.10, 0.47)
	Obese	0.59 (0.15, 1.02)	0.31 (-0.14, 0.76)	0.28 (-0.15, 0.72)
Study Year	2009-10 (reference)			
	2010-11	0.11 (-0.47, 0.70)	0.23 (-0.34, 0.80)	0.17 (-0.37, 0.72)
	2011-2	0.30 (-0.06, 0.67)	0.13 (-0.24, 0.51)	0.30 (-0.05, 0.67)
Baseline sickness absence		0.09 (0.06, 0.11)		**0.07 (0.05, 0.09)**

Negative binomial coefficients shown; CI = confidence interval; PCS-8 = Physical Component Summary score derived from the Short Form 8 Questionnaire; MCS-8 = Mental Component Summary score derived from the Short Form 8 Questionnaire; physical activity is categorised using the Cambridge Physical Activity Index; weight status is categorised using body mass index; study year refers to the time period when data were collected; Model A is adjusted for gender, age, degree, home to work distance, physical limitation, physical activity, weight status and study year; Model B is adjusted for gender, age, degree, home to work distance, physical limitation, physical activity, weight status, study year and baseline sickness absence; bold indicates significant results (p < 0.05); Study undertaken in Cambridge, UK (2009-12).

**Table 4:** Associations of change in weekly cycle commuting time with change in PCS-8, MCS-8 and sickness absence (n = 801).

		Unadjusted	Model A	Model B
		Co-efficient (95% CI)	Co-efficient (95% CI)	Co-efficient (95% CI)
**Physical Wellbeing (PCS-8)**	No change (reference)			
	Increase (n = 183)	0.62 (-0.54, 1.79)	0.94 (-0.22, 2.11)	1.01 (-0.05, 2.07)
	Decrease (n = 223)	-0.49 (-1.58, 0.60)	-0.47 (-1.59, 0.64)	-0.31 (-1.33, 0.69)
**Mental Wellbeing (MCS-8)**	No change (reference)			
	Increase (n = 183)	-0.31 (-1.68, 1.06)	0.20 (-1.26, 1.65)	0.69 (-0.59, 1.97)
	Decrease (n = 223)	-0.51 (-1.79, 0.77)	-0.11 (-1.51, 1.29)	-0.18 (-1.41, 1.05)
**Sickness Absence (days)**	No change (reference)			
	Increase (n = 183)	-0.14 (-1.20, 0.90)	-0.40 (-1.51, 0.72)	-0.37 (-1.33, 0.59)
	Decrease (n = 223)	0.23 (-0.75, 1.21)	0.03 (-1.03, 1.10)	-0.14 (-1.06, 0.79)

Linear regression coefficients shown; CI = confidence interval; PCS-8 = Physical Component Summary score derived from the Short Form 8 Questionnaire; MCS-8 = Mental Component Summary score derived from the Short Form 8 Questionnaire; Model A is adjusted for gender, age, degree, home to work distance, physical limitation, physical activity, weight status and study year; Model B is adjusted for gender, age, degree, home to work distance, physical limitation, physical activity, weight status, study year and appropriate baseline health index (baseline PCS-8 for PCS-8 model, baseline MCS-8 for MCS-8 model or baseline sickness absence for sickness absence model); Study undertaken in Cambridge, UK (2009-12).

## Appendix A Supplementary data

Appendix: supplementary information

Supplementary material: SF-8 sample questionnaire
