# Supplementary material for: Longitudinal associations of active commuting with wellbeing and sickness absence
Source: Prev Med. 2016 Mar;84:19–26. doi: 10.1016/j.ypmed.2015.12.010 (PMC4766368; doi:10.1016/j.ypmed.2015.12.010)
Supplement: Supplementary file 2 — Appendix: supplementary information [file mmc2.docx]

**Appendix**

**Table A1: Characteristics of participants included and excluded from the analyses**

|  | **Included (n=801)** | **Excluded** |
| --- | --- | --- |
|  | **N (%)** | **N (%)** |
| **Gender** |  |  |
| Female | 558 (69.7) | 414 (69.0) |
| Male | 243 (30.3) | 186 (31.0) |
| **Age** |  |  |
| Median (IQR) | 43.3 (33.7-52.2) | 38.5 (31.0-48.1) [n=607] |
| 16-29 years | 106 (13.2) | 133 (21.9) |
| 30-39 years | 220 (27.5) | 196 (32.3) |
| 40-49 years | 210 (26.2) | 153 (25.2) |
| 50-59 years | 199 (24.8) | 90 (14.8) |
| ≥60 years | 66 (8.2) | 35 (5.7) |
| **Highest educational qualification** |  |  |
| Less than degree | 239 (39.8) | 188 (31.6) |
| Bachelor or higher | 562 (70.2) | 406 (68.4) |
| **Deprivation quintile** |  |  |
| 1 (= least deprived) | 364 (45.4) | 266 (41.3) |
| 2 | 235 (29.3) | 187 (31.1) |
| 3 | 104 (13.0) | 91 (15.1) |
| 4 | 92 (11.5) | 55 (9.2) |
| 5 (= most deprived) | 6 (0.7) | 2 (0.3) |
| **Weight status** |  |  |
| Normal or underweight | 524 (65.4) | 351 (60.0) |
| Overweight | 205 (25.6) | 168 (28.7) |
| Obese | 72 (9.0) | 66 (11.2) |
| **PCS-8 score** |  |  |
| Median (IQR) | 55.5 (51.6-58.0) | 55.0 (51.5-57.7) [n=602] |
| **MCS-8 score** |  |  |
| Median (IQR) | 52.5 (48.5-57.5) | 52.1 (45.8-56.4) [n=602] |
| **Sickness Absence (days per year)** |  |  |
| Median (IQR) | 1 (0-4) | 2 (0-4) [n=584] |
| **Disability** |  |  |
| Yes | 30 (3.7) | 21 (3.4) |
| No | 771 (96.3) | 589 (96.6) |
| **Home to work distance** |  |  |
| Median (IQR) | 8.0 (4.8-22.5) | 8.0 (3.2-20.9) [n=608] |
| 0-9.99 km | 471 (58.8) | 348 (57.2) |
| 10-19.99 km | 117 (14.6) | 104 (17.1) |
| ≥20 km | 213 (26.6) | 156 (25.7) |
| **Physical activity index** |  |  |
| Inactive | 24 (3.0) | 20 (3.3) |
| Moderately inactive | 220 (27.5) | 165 (27.2) |
| Moderately active | 245 (30.6) | 200 (33.0) |
| Active | 312 (39.0 | 221 (36.4) |
| **Weekly time cycling to work (minutes)** |  |  |
| Median (IQR) | 56 (0-150) | 40 (0-150) [n=610] |
| **Weekly time walking to work (minutes)** |  |  |
| Median (IQR) | 0 (0-10) | 0 (0-30) [n=605] |

IQR=Interquartile range; PCS-8 = Physical Component Summary score derived from the Short Form 8 Questionnaire; MCS-8 = Mental Component Summary score derived from the Short Form 8 Questionnaire; deprivation quintile is based on national quintiles of deprivation ranked using the Index of Multiple Deprivation 2010 score for the Lower Super Output Area (assigned based on postcode or residence).

**Table A2: Associations between maintenance of walking to work and wellbeing (n=659)**

|  |  | **Physical Wellbeing** | | | **Mental Wellbeing** | | |
| --- | --- | --- | --- | --- | --- | --- | --- |
|  |  | **Unadjusted** | **Model A** | **Model B** | **Unadjusted** | **Model A** | **Model B** |
|  |  | Co-efficient  (95% CI) | Co-efficient  (95% CI) | Co-efficient  (95% CI) | Co-efficient  (95% CI) | Co-efficient  (95% CI) | Co-efficient  (95% CI) |
| Walking | None (reference) |  |  |  |  |  |  |
|  | Some | -0.18 (-1.39, 1.02) | -0.18 (-1.39, 1.03) | -0.15 (-1.27, 0.97) | -1.50 (-3.01, 0.02) | -1.36 (-2.86, 0.14) | -0.65 (-1.99, 0.68) |
| Gender | Male (reference) |  |  |  |  |  |  |
|  | Female | -0.34 (-1.40, 0.71) | -0.46 (-1.53, 0.61) | -0.46 (-1.46, 0.52) | **-1.78 (-3.11, 0.45)** | -1.13 (-1.25, 1.57) | -0.86 (-2.03, 0.32) |
| Age | 16-29 years (reference) |  |  |  |  |  |  |
|  | 30-39 years | 0.64 (-1.01, 2.31) | 0.72 (-0.94, 2.40) | 1.13 (-0.41, 2.68) | **3.36 (1.31, 5.42)** | **3.29 (1.22, 5.36)** | **1.99 (0.15, 3.84)** |
|  | 40-49 years | -0.24 (-1.91, 1.42) | -0.24 (-1.93, 1.44) | 0.14 (-1.42, 1.71) | **4.64 (2.59, 6.71)** | **4.64 (2.55, 6.73)** | **3.45 (1.59, 5.32)** |
|  | 50-59 years | -0.87 (-2.55, 0.81) | -0.81 (-2.53, 0.91) | -0.50 (-2.10, 1.10) | **5.31 (3.22, 7.39)** | **5.30 (3.17, 7.44)** | **3.81 (1.90, 5.72)** |
|  | ≥60 years | -1.62 (-3.83, 0.59) | -1.47 (-3.72, 0.77) | -0.97 (-3.05, 1.11) | **6.33 (3.58, 9.07)** | **6.67 (3.89, 9.45)** | **4.26 (1.75, 6.75)** |
| Higher Degree | No (reference) |  |  |  |  |  |  |
|  | Yes | -0.36 (-1.44, 0.72) | -0.89 (-2.04, 0.25) | **-1.24 (-2.30, -0.18)** | 0.16 (-1.21, 1.52) | 0.16 (-1.26, 1.58) | -0.11 (-1.37, 1.15) |
| Home to work distance | 0-9.99 km (reference) |  |  |  |  |  |  |
|  | 10-19.99 km | 0.34 (-1.05, 1.74) | 0.62(-0.78, 2.31) | 0.62 (-0.68, 1.92) | 1.25 (-0.51, 3.02) | 0.54 (-1.20, 2.29) | 0.67 (-0.87, 2.23) |
|  | ≥20 km | -0.58 (-1.73, 0.58) | -0.59 (-1.79, 0.61) | -0.70 (-1.81, 0.41) | 0.58 (-0.88, 2.03) | 0.55 (-0.93, 2.04) | 0.90 (-0.42, 2.22) |
| Disability | No (reference) |  |  |  |  |  |  |
|  | Yes | -**5.38 (-7.81, -2.95)** | -**5.22 (-7.69, -2.77)** | 2.59 (-0.13, 5.31) | **-4.42 (-7.52, -1.33)** | **-3.93 (-6.99, -0.89)** | **-3.31 (-6.03, -0.61)** |
| Physical Activity | Inactive (reference) |  |  |  |  |  |  |
|  | Moderately inactive | 1.88 (-1.29, 5.06) | 1.15 (-2.06, 4.36) | -0.12 (-3.11, 2.86) | **6.46 (2.47, 10.5)** | **6.61 (2.63, 10.6)** | **3.91 (0.35, 7.47)** |
|  | Moderately active | 2.90 (-0.26,6.05) | 1.94 (-1.28,5.18) | 0.37 (-2.63, 3.39) | **6.76 (2.80, 10.7)** | **6.99 (2.99, 11.0)** | **3.90 (0.32, 7.50)** |
|  | Active | 2.89 (-0.23, 6.02) | 1.87 (-1.33, 5.07) | 0.61 (-2.35, 3.59) | **7.25 (3.32, 11.2)** | **7.42 (3.45, 11.4)** | **3.79 (0.23, 7.36)** |
| Weight status | Low or healthy (reference) |  |  |  |  |  |  |
|  | Overweight | -0.71 (-1.84, 0.43) | -0.25 (-1.42, 0.93) | -0.28 (-1.37, 0.86) | -0.55 (-1.99, 0.90) | -1.18 (-2.63, 0.28) | -0.94 (-2.24, 0.36) |
|  | Obese | **-1.92 (-3.62, -0.22)** | **-1.65 (-3.42, -0.13)** | -0.68 (-2.34, 0.97) | 0.03 (-2.11, 2.19) | -0.02 (-2.20, 2.27) | 0.10 (-1.85, 2.04) |
| Study Year | 2009-10 (reference) |  |  |  |  |  |  |
|  | 2010-11 | 0.05 (-2.09, 2.19) | -1.11 (-3.34, 1.12) | -1.02 (-3.09, 1.05) | 1.09 (-1.75, 3.94) | 1.16 (-1.59, 3.93) | 0.42 (2.04, 2.88) |
|  | 2011-2 | -0.74 (-2.10, 0.60) | -0.94 (-2.41, 0.52) | -1.04 (-2.40, 0.33) | -0.58 (-2.38, 1.21) | -0.79 (-2.11, 1.02) | -0.68 (-2.30, 0.94) |
| Baseline health |  | **0.42 (0.36, 0.50)** |  | **0.47 (0.38, 0.55)** | 0.52 (0.45, 0.59) |  | **0.48 (0.41, 0.55)** |

Linear regression coefficients shown; CI=confidence interval; PCS-8 = Physical Component Summary score derived from the Short Form 8 Questionnaire; MCS-8 = Mental Component Summary score derived from the Short Form 8 Questionnaire; physical activity is categorised using the Cambridge Physical Activity Index; weight status is categorised using body mass index; study year refers to the time period when data were collected; Model A is adjusted for gender, age, degree, home to work distance, physical limitation, physical activity, weight status and study year; Model B is adjusted for gender, age, degree, home to work distance, physical limitation, physical activity, weight status, study year and baseline wellbeing (baseline PCS-8 for PCS-8 model or baseline MCS-8 for MCS-8 model); bold indicates significant results (p<0.05); Study undertaken in Cambridge, UK (2009-12).

**Table A3: Associations between maintenance of walking to work and sickness absence (n=659)**

|  |  | **Unadjusted** | **Model A** | **Model B** |
| --- | --- | --- | --- | --- |
|  |  | Co-efficient  (95% CI) | Co-efficient  (95% CI) | Co-efficient  (95% CI) |
| Walking | None (reference) |  |  |  |
|  | Some | -0.02 (-0.35, 0.31) | 0.20 (-0.13, 0.53) | 0.12 (-0.19, 0.43) |
| Gender | Male (reference) |  |  |  |
|  | Female | 0.45 (0.16, 0.75) | 0.23 (-0.06, 0.53) | 0.27 (-0.01, 0.54) |
| Age | 16-29 years (reference) |  |  |  |
|  | 30-39 years | **-1.05 (-1.49, 0.62)** | **-0.84 (-1.28, -0.40)** | **-0.79 (-1.20, -0.37)** |
|  | 40-49 years | **-1.09 (-1.53, 0.65)** | **-0.97 (-1.42, -0.53)** | **-0.87 (-1.29, -0.44)** |
|  | 50-59 years | **-0.89 (-1.33, -0.44)** | **-0.81 (-1.27, -0.35)** | **-0.73 (1.18, -0.29)** |
|  | ≥60 years | **-1.10 (-1.68, -0.51)** | **-0.98 (-1.57, -0.38)** | **-0.66 (-1.23, -0.09)** |
| Higher Degree | No (reference) |  |  |  |
|  | Yes | **-0.10 (-0.40, 0.19)** | -0.04 (-0.34, 0.27) | 0.08 (-0.21, 0.36) |
| Home to work distance | 0-9.99 km (reference) |  |  |  |
|  | 10-19.99 km | -0.15 (-0.53, 0.24) | 0.12 (-0.25, 0.51) | 0.07 (-0.30, 0.44) |
|  | ≥20 km | **0.03 (-0.29, 0.34)** | 0.11 (-0.21, 0.43) | 0.07 (-0.24, 0.37) |
| Disability | No (reference) |  |  |  |
|  | Yes | **1.06 (0.41, 1.72)** | **1.01 (0.39, 1.63)** | 0.45 (-0.16, 1.06) |
| Physical Activity | Inactive (reference) |  |  |  |
|  | Moderately inactive | -1.82 (-2.64, -1.01) | -0.82 (-1.69, 0.04) | 0.35 (-0.53, 1.22) |
|  | Moderately active | -1.77 (-2.58, -0.96) | -1.02 (-1.87, -0.16) | 0.23 (-0.65, 1.10) |
|  | Active | -1.73 (-2.53, -0.93) | -0.82 (-1.68, 0.02) | 0.27 (-0.60, 1.13) |
| Weight status | Normal (reference) |  |  |  |
|  | Overweight | -0.02 (-0.33, 0.29) | 0.11 (-0.20, 0.42) | 0.12 (-0.18, 0.42) |
|  | Obese | 0.99 (0.54, 1.43) | 0.34 (-0.14, 0.82) | 0.22 (-0.24, 0.69) |
| Study Year | 1 (reference) |  |  |  |
|  | 2 | 0.09 (-0.52, 0.69) | 0.19 (-0.40, 0.77) | 0.13 (-0.43, 0.70) |
|  | 3 | 0.93 (0.56, 1.30) | 0.63 (0.24, 1.03) | 0.77 (0.40, 1.14) |
| Baseline sickness absence |  | **0.09 (0.07, 1.22)** |  | **0.07 (0.05, 0.10)** |

Negative binomial coefficients shown; CI=confidence interval; PCS-8 = Physical Component Summary score derived from the Short Form 8 Questionnaire; MCS-8 = Mental Component Summary score derived from the Short Form 8 Questionnaire; physical activity is categorised using the Cambridge Physical Activity Index; weight status is categorised using body mass index; study year refers to the time period when data were collected; Model A is adjusted for gender, age, degree, home to work distance, physical limitation, physical activity, weight status and study year; Model B is adjusted for gender, age, degree, home to work distance, physical limitation, physical activity, weight status, study year and baseline sickness absence; bold indicates significant results (p<0.05); Study undertaken in Cambridge, UK (2009-12).

**Table A4: Associations of changes in weekly walk commuting time with change in PCS-8, MCS-8 and sickness absence (n=801)**

|  |  | **Unadjusted** | **Model A** | **Model B** |
| --- | --- | --- | --- | --- |
|  |  | Co-efficient (95% CI) | Co-efficient (95% CI) | Co-efficient (95% CI) |
| **Physical Wellbeing (PCS-8)** | No change  (reference) |  |  |  |
|  | Increase (n=139) | -0.36 (-1.61, 0.88) | -0.07 (-1.26, 1.13) | -0.21 (-1.29, 0.88) |
|  | Decrease (n=126) | 0.02 ((-1.29, 1.31) | -0.07 (-1.31, 1.15) | -0.22 (-1.34, 0.90) |
|  |  |  |  |  |
| **Mental Wellbeing (MCS-8)** | No change  (reference) |  |  |  |
|  | Increase (n=139) | 0.94 (-0.52, 2.39) | 0.93 (-0.56, 2.42) | 0.38 (-0.93, 1.69) |
|  | Decrease (n=126) | -0.25 (-1.78, 1.26) | -0.14 (-1.68, 1.39) | -0.44 (-1.78, 0.91) |
|  |  |  |  |  |
| **Sickness Absence (days)** | No change  (reference) |  |  |  |
|  | Increase (n=139) | -0.87 (-1.98, 0.25) | -0.65 (-1.79, 0.48) | -0.33 (-1.31, 0.66) |
|  | Decrease (n=126) | -0.50 (-1.66, 0.66) | -0.41 (-1.58, 0.76) | 0.20 (-0.81, 1.22) |

Linear regression coefficients shown; CI=confidence interval; PCS-8 = Physical Component Summary score derived from the Short Form 8 Questionnaire; MCS-8 = Mental Component Summary score derived from the Short Form 8 Questionnaire; Model A is adjusted for gender, age, degree, home to work distance, physical limitation, physical activity, weight status and study year; Model B is adjusted for gender, age, degree, home to work distance, physical limitation, physical activity, weight status, study year and appropriate baseline health index (baseline PCS-8 for PCS-8 model, baseline MCS-8 for MCS-8 model or baseline sickness absence for sickness absence model); Study undertaken in Cambridge, UK (2009-12).

**Table A5: Associations of large changes in weekly cycle commuting time (≥ 50 minutes per week) with change in PCS-8, MCS-8 and sickness absence**

|  |  | **Unadjusted** | **Model A** | **Model B** |
| --- | --- | --- | --- | --- |
|  |  | Co-efficient (95% CI) | Co-efficient (95% CI) | Co-efficient (95% CI) |
| **Physical Wellbeing (PCS-8)** | No change or change less than 50 minutes  (reference) |  |  |  |
|  | Large increase (n=114) | 0.70 (-0.65, 2.05) | 0.80 (-0.50, 2.10) | 1.10 (-0.08, 2.28) |
|  | Large decrease (n=158) | -0.03 (-1.21, 1.15) | -0.37 (-1.52, 0.79) | -0.13 (-1.18, 0.91) |
|  |  |  |  |  |
| **Mental Wellbeing (MCS-8)** | No change or change less than 50 minutes  (reference) |  |  |  |
|  | Large increase (n=114) | -0.28 (-1.86, 1.30) | 0.17 (-1.44, 1.79) | 0.60 (-0.82, 2.02) |
|  | Large decrease (n=158) | -0.61 (-2.00, 0.78) | -0.25 (-1.69, 1.18) | -0.47 (-1.73, 0.79) |
|  |  |  |  |  |
| **Sickness Absence (days)** | No change or change less than 50 minutes  (reference) |  |  |  |
|  | Large Increase (n=114) | 0.21 (-0.99, 1.42) | 0.19 (-1.05, 1.43) | -0.34 (-1.41, 0.73) |
|  | Large decrease (n=158) | 0.96 (-0.10, 2.02) | 0.93 (-0.17, 2.03) | 0.46 (-0.49, 1.41) |

Linear regression coefficients shown; CI=confidence interval; PCS-8 = Physical Component Summary score derived from the Short Form 8 Questionnaire; MCS-8 = Mental Component Summary score derived from the Short Form 8 Questionnaire; Model A is adjusted for gender, age, degree, home to work distance, physical limitation, physical activity, weight status and study year; Model B is adjusted for gender, age, degree, home to work distance, physical limitation, physical activity, weight status, study year and appropriate baseline health index (baseline PCS-8 for PCS-8 model, baseline MCS-8 for MCS-8 model or baseline sickness absence for sickness absence model); Large increase defined as increase of more than 50 minutes per week and a large decrease defined as a decrease of more than 50minutes per week; Total sample size: n=801; Study undertaken in Cambridge, UK (2009-12).

**Table A6: Associations of large changes in weekly walk commuting time (≥ 50 minutes per week) with change in PCS-8, MCS-8 and sickness absence (n=801)**

|  |  | **Unadjusted** | **Model A** | **Model B** |
| --- | --- | --- | --- | --- |
|  |  | Co-efficient (95% CI) | Co-efficient (95% CI) | Co-efficient (95% CI) |
| **Physical Wellbeing (PCS-8)** | No change or change less than 50 minutes  (reference) |  |  |  |
|  | Large Increase (n=114) | -0.23 (-1.76, 1.30) | -0.02 (-1.48, 1.45) | -0.06 (-1.39, 1.27) |
|  | Large Decrease (n=158) | -0.35 (-1.84, 1.15) | -0.33 (-1.76, 1.10) | -0.53 (-1.82, 0.77) |
|  |  |  |  |  |
| **Mental Wellbeing (MCS-8)** | No change or change less than 50 minutes  (reference) |  |  |  |
|  | Increase (n=82) | 1.63 (-0.16, 3.43) | 1.74 (-0.07, 3.57) | 1.15 (-0.45, 2.74) |
|  | Decrease (n=87) | -0.24 (-1.99, 1.51) | 0.07 (-1.69, 1.85) | -0.34 (-1.90, 1.22) |
|  |  |  |  |  |
| **Sickness Absence (days)** | No change or change less than 50 minutes  (reference) |  |  |  |
|  | Increase (n=82) | -0.75 (-2.12, 0.63) | -0.60 (-1.99, 0.80) | -0.53 (-1.73, 0.68) |
|  | Decrease (n=87) | -0.97 (-2.31, 0.37) | -0.96 (-2.33, 0.40) | 0.22 (-1.40, 0.96) |

Linear regression coefficients shown; CI=confidence interval; PCS-8 = Physical Component Summary score derived from the Short Form 8 Questionnaire; MCS-8 = Mental Component Summary score derived from the Short Form 8 Questionnaire; Model A is adjusted for gender, age, degree, home to work distance, physical limitation, physical activity, weight status and study year; Model B is adjusted for gender, age, degree, home to work distance, physical limitation, physical activity, weight status, study year and appropriate baseline health index (baseline PCS-8 for PCS-8 model, baseline MCS-8 for MCS-8 model or baseline sickness absence for sickness absence model); Large increase defined as increase of more than 50 minutes per week and a large decrease defined as a decrease of more than 50minutes per week; Total sample size: n=801; Study undertaken in Cambridge, UK (2009-12).
