# Supplementary material for: Longitudinal associations of active commuting with wellbeing and sickness absence
Source: Prev Med. 2016 Mar;84:19–26. doi: 10.1016/j.ypmed.2015.12.010 (PMC4766368; doi:10.1016/j.ypmed.2015.12.010)
Supplement: Supplementary file 3 — Supplementary material: SF-8 sample questionnaire [file mmc3.pdf]

Your Health

– and –

Well-Being

SAMPLE - NOT FOR DISTRIBUTION

This survey asks for your views about your health. This information will help keep track of how you feel and how well you are able to do your usual activities.

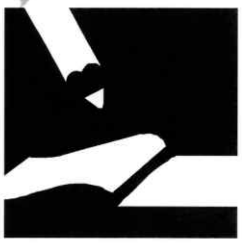

Thank you for completing these questions!

SF-8™ Health Survey
Copyright © 1998,1999 QualityMetric Incorporated. All rights reserved.
SF-8 Standard - UK (English Version 1.0)

1. Overall, how would you rate your health during the past 4 weeks? [Mark with a ☑ in the one box that best describes your answer.]

Excellent	Very good	Good	Fair	Poor	Very poor
τ	τ	τ	τ	τ	τ
☐ 1	☐ 2	☐ 3	☐ 4	☐ 5	☐ 6

2. During the past 4 weeks, how much did physical health problems limit your usual physical activities (walking, climbing stairs)?

Not at all	Very little	Somewhat	Quite a lot	Could not do physical activities
τ	τ	τ	τ	τ
☐ 1	☐ 2	☐ 3	☐ 4	☐ 5

3. During the past 4 weeks, how much difficulty did you have doing your daily work, both inside and outside the home, because of your physical health?

None at all	A little bit	Some	Quite a lot	Could not do daily work
τ	τ	τ	τ	τ
☐ 1	☐ 2	☐ 3	☐ 4	☐ 5

SAMPLE - NOT FOR DISTRIBUTION

Copyright © 1998,1999 QualityMetric Incorporated. All rights reserved.
SF-8 Standard - UK (English Version 1.0)
No part of the Health Survey may be reproduced or transmitted in any form or by any means – electronic, mechanical; including photocopy, recording or any information storage or retrieval system – without permission of the copyright holder
Requests for permission to reproduce the SF-8™ Health Survey should be sent to Optum, 24 Albion Rd., Lincoln, RI 02865 or https://www.optum.com/optum-outcomes.html.

4. How much bodily pain have you had during the past 4 weeks?

None	Very mild	Mild	Moderate	Severe	Very severe
τ	τ	τ	τ	τ	τ
☐ 1	☐ 2	☐ 3	☐ 4	☐ 5	☐ 6

5. During the past 4 weeks, how much energy did you have?

Very much	Quite a bit	Some	A little	None
τ	τ	τ	τ	τ
☐ 1	☐ 2	☐ 3	☐ 4	☐ 5

6. During the past 4 weeks, how much did your physical health or emotional problems limit your usual social activities with family or friends?

Not at all	Very little	Somewhat	Quite a lot	Could not do social activities
τ	τ	τ	τ	τ
☐ 1	☐ 2	☐ 3	☐ 4	☐ 5

SAMPLE - NOT FOR DISTRIBUTION

Copyright © 1998,1999 QualityMetric Incorporated. All rights reserved.
SF-8 Standard - UK (English Version 1.0)
No part of the Health Survey may be reproduced or transmitted in any form or by any means – electronic, mechanical; including photocopy, recording or any information storage or retrieval system – without permission of the copyright holder
Requests for permission to reproduce the SF-8™ Health Survey should be sent to Optum, 24 Albion Rd., Lincoln, RI 02865 or https://www.optum.com/optum-outcomes.html.

7. During the <u>past 4 weeks</u>, how much have you been bothered by <u>emotional problems</u> (such as feeling anxious, depressed or irritable)?

Not at all	Slightly	Moderately	Quite a lot	Extremely
τ	τ	τ	τ	τ
☐ 1	☐ 2	☐ 3	☐ 4	☐ 5

8. During the <u>past 4 weeks</u>, how much did personal or emotional problems keep you from doing your usual work, studies, or other daily activities?

Not at all	Very little	Somewhat	Quite a lot	Could not do daily activities
τ	τ	τ	τ	τ
☐ 1	☐ 2	☐ 3	☐ 4	☐ 5

SAMPLE - NOT FOR DISTRIBUTION

Thank you for completing these questions!

Copyright © 1998,1999 QualityMetric Incorporated. All rights reserved.
SF-8 Standard - UK (English Version 1.0)
No part of the Health Survey may be reproduced or transmitted in any form or by any means – electronic, mechanical; including photocopy, recording or any information storage or retrieval system – without permission of the copyright holder
Requests for permission to reproduce the SF-8™ Health Survey should be sent to Optum, 24 Albion Rd., Lincoln, RI 02865 or https://www.optum.com/optum-outcomes.html.
